# 359. Clinical implications of SARS-CoV-2 PCR presumptive positive results

**DOI:** 10.1093/ofid/ofab466.560

**Published:** 2021-12-04

**Authors:** Takeru Yamamoto, Masako Mizusawa

**Affiliations:** University of Missouri-Kansas City, Kansas City, Missouri

## Abstract

**Background:**

Severe acute respiratory syndrome coronavirus 2 (SARS-CoV-2) reverse transcriptase (RT) polymerase chain reaction (PCR) testing is a cornerstone of coronavirus disease 2019 (COVID-19) diagnostics. A number of RT-PCR tests are currently available with different combinations of target genes including the pan-Sarbecovirus E gene target. When the E gene target is positive and the SARS-CoV-2 specific target is negative, the result is reported as presumptive positive which may indicate 1) a sample at concentrations near or below the limit of detection, 2) a mutation in the SARS-CoV-2 specific target, 3) infection with some other Sarbecovirus or 4) other factors. However, what the presumptive positive results mean clinically and whether they should be treated in the same way as the positive results are unknown.

**Methods:**

We conducted a retrospective review of electronic health records for patients who had the presumptive positive results by Xpert Xpress SARS-CoV-2 (Cepheid, Sunnyvale, CA) or Cobas SARS-CoV-2 (Roche Molecular Systems, Branchburg, NJ) at Truman Medical Center from April 13, 2020 to December 31, 2021. Demographics, comorbidities, symptoms, laboratory data, radiographic data, clinical course and COVID-19 related complications were recorded and analyzed.

**Results:**

During the study period, 85,267 SARS-CoV-2 RT-PCR tests were performed and 253 (0.3%) presumptive positive results were reported for 243 patients. Symptom information were available for 178 patients and 70% of them were symptomatic at the time of testing. Only 2 patients were admitted for COVID-19 pneumonia with the presumptive positive results. Both of them had low oxygen requirement during hospitalization and were discharged with stable conditions.

**Conclusion:**

Symptomatic COVID-19 patients who presented with presumptive positive results by Xpert Xpress SARS-CoV-2 or Cobas SARS-CoV-2 had generally mild disease and rarely required hospitalization for COVID-19.

**Disclosures:**

**All Authors**: No reported disclosures

